# Transglutaminase 2: The Maestro of the Oncogenic Mediators in Renal Cell Carcinoma

**DOI:** 10.3390/medsci7020024

**Published:** 2019-02-06

**Authors:** Ayca Ece Nezir, Burge Ulukan, Dilek Telci

**Affiliations:** Department of Genetics and Bioengineering, Faculty of Engineering, Yeditepe University, Kayisdagi Cad., Istanbul 34755, Turkey; ayca.nezir@gmail.com (A.E.N.); ulukanburge@gmail.com (B.U.)

**Keywords:** Transglutaminase 2 (TG2), Renal Cell Carcinoma (RCC), metastasis, invasion, epithelial mesenchymal transition

## Abstract

Transglutaminase 2 (TG2) is a multifunctional crosslinking enzyme that displays transamidation, protein disulfide isomerase, protein kinase, as well as GTPase and ATPase activities. TG2 can also act as an adhesion molecule involved in the syndecan and integrin receptor signaling. In recent years, TG2 was implicated in cancer progression, survival, invasion, migration, and stemness of many cancer types, including renal cell carcinoma (RCC). Von Hippel-Lindau mutations leading to the subsequent activation of Hypoxia Inducible Factor (HIF)-1-mediated signaling pathways, survival signaling via the PI3K/Akt pathway resulting in Epithelial Mesenchymal Transition (EMT) metastasis and angiogenesis are the main factors in RCC progression. A number of studies have shown that TG2 was important in HIF-1- and PI3K-mediated signaling, VHL and p53 stabilization, glycolytic metabolism and migratory phenotype in RCC. This review focuses on the role of TG2 in the regulation of molecular pathways nurturing not only the development and propagation of RCC, but also drug-resistance and metastatic potential.

## 1. Introduction

Renal cancer or renal cell carcinoma (RCC) is a subtype of kidney cancer with a high mortality rate; it is listed among the top ten leading causes of cancer-related death [1]. Although curative surgical resection can be amenable in early detection, RCC is clinically silent for much of its natural course, and in most cases, patients have already developed metastases at the time of diagnosis. Moreover, traditional approaches such as the determination of tumor stage, nuclear grade and histological tumor necrosis remain poor for the assessment of RCC prognosis, and the existing lines of treatment cannot provide a long-term disease-free survival for patients [2,3].

Transglutaminase 2 (TG2) is the ubiquitously-expressed member of the transglutaminase family of enzymes, which can catalyze the calcium-dependent crosslinking of its target molecules [4,5]. This transamidation reaction results in the post-translational modification (PTM) of its target proteins. The enzyme activity of TG2 is mediated by its reversible conformational changes. While the catalytic domain is available for substrate interaction in its Ca2+-bound form, the binding of guanosine nucleotides (GTP/GDP) to TG2 shifts the protein to its closed, catalytically-inactive state [6,7,8,9,10,11,12,13,14]. In the closed state, TG2 acts as an atypical GTPase molecule, and plays a role as a transducer protein in phospholipase Cδ1 (PLCδ1) signaling pathway [15]. Furthermore, TG2 has protein kinase, protein disulfide isomerase (PDI) [16,17,18], and ATP-binding/hydrolyzing activities [19,20,21,22,23]. TG2 can also act as an adhesion molecule via its interactions with fibronectin (FN), syndecan-4 (SDC4) and integrin beta-1 (ITGB1) [24,25,26].

Transglutaminase 2 expression and activity have been implicated in the inflammatory processes and diseases, including cancer [27]. Transamidating activity of TG2 has a tumor-suppressive function in healthy cells by stabilizing extracellular matrix (ECM), but an oncogenic potential in malignant cells [28,29]. Elevated TG2 expression has been demonstrated in a number of cancers such as pancreatic [30], breast [31], melanoma [32], ovary [33], lung [34], and lastly RCC [35,36], in association with cancer progression, drug resistance, metastatic spread, and poor patient survival. Although many studies revealed the multi-faced biological activities of TG2 in cancers with epithelial origin, there is still need to mechanistically dissect the upstream and downstream regulators of prometastatic TG2 at a molecular level in RCC development. In this review, we focus on the role of TG2 in the modulation of three intertwined molecular signaling mechanisms including NF-κB/HIF, PI3K/Akt/mTOR signaling and p53 pathway.

## 2. Significance of Transglutaminase 2 in Renal Cell Carcinoma

Renal cell carcinoma consists of a heterogeneous group of tumors derived from various parts of the nephron, possessing distinct genetic and histological characteristics [37,38,39,40]. Major subtypes with high incidence are clear cell RCC (ccRCC), papillary RCC (pRCC) and chromophobe RCC (chRCC). The complexity of RCC makes it difficult to obtain a durable complete response from any treatment method. Surgical resection benefits RCC patients with locally advanced tumors; however, most of the patients suffer recurrent metastases [41,42]. Interleukin-2 and interferon have been the standard care for patients with metastatic RCC (mRCC) for more than 20 years, but the overall survival has not improved significantly [43]. Reports show that these agents benefit only a very select group of patients with good prognoses. While interleukin-2 and interferon therapies can still be utilized in a case-specific manner, targeting defined molecular pathways in RCC has become more prevalent for the treatment [44]. In this respect, vascular endothelial growth factor (VEGF) and mechanistic target of rapamycin (mTOR) pathways have been established as relevant targets in RCC, since most patients suffer from the aberrant activation of these pathways due to genetic and/or epigenetic alterations. Although several VEGF inhibitors (sunitinib, pazopanib, sorafenib, bevacizumab) and mTOR inhibitors (temsirolimus, everolimus) have overall increased the rate of disease-free survival rate in mRCC treatments, the lack of complete tumor remission suggested that further therapeutic advances are still required [42].

Recent efforts to identify the molecular mechanisms in tumor oncogenesis and metastasis were based on genome-wide studies of non-coding RNAs (ncRNAs). Small ncRNA molecules called microRNAs (miRNAs) can regulate gene expression either by repressing the translation of their mRNA targets, or by cleaving them in a sequence-specific manner [45]. In cancer biology, miRNAs can function as oncogenes by repressing the tumor suppressors, or act as tumor suppressors by negatively regulating the oncogenes [46]. Accumulating evidence suggests that the alterations in miRNA levels can be responsible for the acquisition of all hallmarks of cancer, including the self-sustained cell growth and loss of cell cycle control, resistance to apoptosis, tissue invasion and metastasis, angiogenesis, and unlimited replicative potential [47]. miRNA expression signatures and their functional analyses have been carried out in RCC to unravel the suitable therapeutic targets, as well as diagnostic and prognostic biomarkers [48]. By analyzing clear cell type clinical RCC specimens collected by total nephrectomy in comparison to the adjacent non-cancerous tissues, a total of 103 miRNAs were found to be downregulated [49]. Gain-of-function studies in A-498, 786-O, Caki-2 primary and ACHN metastatic RCC cell lines showed that miR-1285 had the strongest inhibitory effect on RCC cell proliferation. Furthermore, the invasion and migration potential of these RCC cells were hindered upon miR-1285 transfection. Among the putative miR-1285 targets identified in A-498 and 786-O cells, TG2 was the only gene that was expressed at significantly higher levels in the clinical specimens, compared to the non-cancerous counterparts. Reduction in cell proliferation, migration, and invasion potential following the loss-of-function studies with TG2-directed siRNA suggested that the preferential downregulation of miR-1285, resulting in the subsequent upregulation of TG2, might favor RCC oncogenesis [49].

## 3. Relevance of Transglutaminase 2 in Epithelial-Mesenchymal Transition and HIF-1 Regulatory Pathway

Epithelial-mesenchymal transition (EMT) is a biological process, where a polarized epithelial cell undergoes biochemical changes that enables it to assume a mesenchymal cell phenotype with an enhanced migratory capacity, invasiveness, and elevated resistance to apoptosis [50]. Although EMT is an essential cellular process during development and wound healing, EMT that is observed in cancer arises as a distinct type that differs from the usual processes by occurrence of an aberrant heterogeneous cell population consisting of both fully and partially transformed cancer cells [51]. This heterogeneous population comprises cancer cells of mesenchymal phenotype, in addition to those that retain several properties of their epithelial origin. Nuclear factor-kappa B (NF-κB) is a crucial player in the induction and maintenance of EMT in transformed epithelial cells [52]. Activation of NF-κB via its dissociation from inhibitory IκB molecules results in its nuclear translocation, followed by the transcriptional activation of its target genes including certain inflammatory cytokines and growth factors, as well as EMT markers such as Zeb1, Zeb2, Snail and Twist transcription factors. The role of TG2 in NF-κB-induced EMT was first reported in breast cancer, which occurs through the non-canonical degradation of IκB molecules due to TG2 mediated PTM [53]. Crosslinking of IκB into polymer form renders IκB molecules incapacitated to bind and inhibit NF-κB resulting in the translocation of the transcription factor into the nucleus. A follow-up study indicated that the GTP-binding/GTPase activity of TG2 was responsible for the induction of EMT and the associated cancer stem cell phenotype in breast cancer [54]. Consistently, TG2 was shown to activate NF-κB in the mouse RCC cell line RenCa, resulting in the subsequent induction of EMT, in a GTP-binding manner [55]. Furthermore, TGF-β1, TNF-α, IL-1 and IL-6, expressions of which depend on NF-κB, are also the transcriptional activators of TG2 [56]. Thus, constitutive activation of NF-κB results in the enhanced transcription of TG2, which in turn further activates NF-κB pathway, building a positive feedback loop. Given that NF-κB is involved in cell proliferation and drug resistance in cancer, TG2 could be a promising new drug target for anticancer therapy [53,57,58].

Aberrant expression of TG2 is associated with the loss of the epithelial features such as type-I E-cadherin expression and cellular polarity, along with upregulation of the mesenchymal markers including N-cadherin, vimentin, fibronectin, and the transcriptional factors Snail1, Zeb1, Zeb2, and Twist1 in breast [59] and several other cancers [55,60,61,62,63,64]. Changes in type-II cadherins, calcium-dependent cell adhesion molecules may also contribute to the acquisition of mesenchymal phenotype by cancer cells. For instance, cadherin-11 was found to be upregulated in prostate cancer cell lines and tissues, as well as in metastatic lesions, while an upregulation was not evident in healthy prostate tissue [65,66]. Based on the alterations of type-II cadherin levels in RCC, it was suggested that these cadherins may also play an important role in RCC tumorigenesis [67]. In RCC, cadherin-6 expression was strongly associated with tumor progression, and was suggested as a prognostic marker [68,69]. A follow-up study screening the expression of type-II cadherins in 16 different RCC cell lines revealed that cadherins 6 and 14 were the most expressed. Although currently there are no studies showing a direct regulation of type-II cadherins by TG2, the involvement of NF-κB in cadherin-6 mediated EMT and metastasis suggests a possible role for TG2 in this signaling axis [70].

The induction of EMT by NF-κB can be promoted through the transcriptional activation of hypoxia inducible factor 1 alpha (HIF-1α), cellular level of which is regulated by Von Hippel-Lindau (VHL) protein [71]. HIF-1α acts as a direct oxygen sensor, being hydroxylated under normoxic conditions. VHL is an E3 ubiquitin ligase that recognizes the hydroxyl group of HIF-1α, targeting it to proteasome-mediated degradation. In case of hypoxia, however, VHL cannot recognize HIF-1α due to the lack of hydroxyl group, and thus, the HIF-1-mediated transcription is activated. The majority of HIF targets involve genes that regulate angiogenesis, glycolysis, and EMT. The VHL gene is frequently mutated or silenced due to hypermethylation in ccRCC [72,73]. This results in the accumulation of HIF-1α and the upregulation of its target genes responsible from the EMT induction. In addition to the genetic and/or epigenetic alterations, VHL levels can be regulated by TG2 in RCC [74]. In RCC cells, TG2 via crosslinking activity can lead to the polymerization of VHL, which results in VHL ubiquitylation and proteasomal degradation. This TG2-mediated inhibition of VHL results in the accumulation of HIF-1α. In addition, TG2 was shown to transcriptionally upregulate HIF-1α via NF-κB by forming a complex with p65 subunit [53]. As HIF-1α is another transcriptional activator of TG2 [75], by mediating the depletion of VHL, TG2 can favor the expression of itself in RCC cells thence act as a tumor-promoter.

## 4. Maintenance of Cancer Characteristics by Transglutaminase 2-Regulated Degradation Pathways

Besides its roles in EMT and angiogenesis HIF-1α can regulate autophagy, a process whereby cells can degrade their own biomolecules and produce monomers for reuse. This pathway is important for the regular turnover of the cellular molecules, as well as for evading pathogenicity. Under stressful conditions where the energy supply is relatively low, autophagy can help cells survive through the recycling of the existing molecules [76]. As a self-degradative system, autophagy also underlies the acquisition of drug resistance in cancer. Autophagy can act as a tumor-suppressive process either by eliminating the carcinogenic elements or triggering a cell death mechanism in support of apoptosis, eradicating the damaged cell itself. However, in advanced cancers, cells can take advantage of this recycling system to self-support where oxygen and nutrient are scarce [77,78,79,80,81,82,83]. HIF-1α can induce autophagy through modulating the release of an important regulator of autophagy, Beclin-1, from its inhibitory complex with Bcl-2 protein [84]. TG2-mediated upregulation and stabilization of HIF-1α in RCC can thus influence the activation of autophagic machinery. In this context, several RCC cell lines displayed enhanced levels of TG2 and autophagy under normal physiological conditions [85].

Usually, misfolded proteins in cells are recognized and ubiquitylated by specific E3 ligases for their subsequent degradation in proteasomes [86,87]. Defects in this ubiquitin-proteasome machinery can lead to the accumulation of misfolded proteins and result in cellular toxicity. Cells can be protected from the toxicity of misfolded proteins through the formation of structures called aggresomes and their clearance by autophagy [88]. Transglutaminase 2 was shown to be involved in the PTM of these high-molecular-weight aggregates, which stabilizes the structure before conveyance to the autophagic machinery [89]. Moreover, TG2 can induce autophagy both in vitro and in vivo, and the ablation of TG2 leads to the impairment of final maturation of autophagolysosomes. The crosslinking of certain cytoskeletal proteins by TG2 may be the underlying mechanism in the targeting of autophagosomes to lysosomes for their fusion [90].

Although TG2-deficient mice display no apparent physiological and developmental defects, it was found that these mice were rather susceptible to apoptotic stress [91]. Since a role for TG2 in the autophagosome formation and the clearance of protein aggregates by autophagy was already suggested [89,90], researchers set out to reveal the possible contribution of TG2-mediated autophagy to the evasion of apoptotic signals [85]. Indeed, the silencing of TG2 in RCC cell lines resulted in a 3- to 10-fold increase in apoptosis due to p53 stabilization, and it was suggested that TG2 played a role in RCC growth by depleting p53 through autophagy. It was further demonstrated that TG2-mediated depletion of p53 was dependent on its crosslinking activity. Caspase 3 and cathepsin D are the two other molecules that are important for the regulation of cell death, and their degradation via autophagy can also be mediated by TG2 crosslinking activity [91,92]. In follow-up in vivo studies, the administration of TG2 inhibitors to RCC xenograft models stabilized the cellular levels of p53 expression and induced apoptosis, suggesting that the inhibition of TG2 may exert a strong therapeutic effect in RCC by inducing elevated levels of autophagy [93,94]. These findings imply that although TG2 may not be the sole inducer of cancer cell proliferation, it contributes to the fine-tuning of survival signals by facilitating the degradation of tumor suppressor proteins.

Transglutaminase 2 not only regulates the autophagic but also the proteasomal degradation of certain molecules. As mentioned, TG2-mediated crosslinking of VHL leads to its ubiquitylation and proteasomal degradation [74]. Another target of TG2 is the phosphatase and tensin homolog (PTEN), which is a negative regulator of the phosphatidylinositol 3-kinase (PI3K) pathway. In this case, TG2 physically associates with PTEN and inhibits its phosphorylation, leading to destabilization of the protein. PTEN is then recognized and ubiquitylated by specific E3 ligases, and is degraded in proteasomes [95]. Depletion of PTEN in cells leads to the activation of PI3K and its downstream mTOR pathway. As the transcription of PTEN can be activated by p53, the degradation of which is mediated by TG2 in RCC, the aberrant activation of mTOR observed in many RCC patients might be defined by a TG2-dependent mechanism.

The intracellular levels of TG2 itself can be regulated through the ubiquitin-dependent degradation system. Carboxyl-terminus of Hsp70-interacting protein (CHIP) promotes the ubiquitylation and subsequent degradation of many tumor-related proteins, including HIF-1α [96], PTEN [97], c-Myc [98], Smad3 [99], and Src-3 [100] through the proteasomal machinery. There are several reports indicating a tumor suppressive function for CHIP in gastric cancer [101], prostate cancer [102], hepatoma [103], glioma [104], and breast cancer [105,106]. Recently, it was found that CHIP mediates TG2 ubiquitylation, and the proteasomal degradation of TG2 is altered in renal cancers due to the downregulation of CHIP [107]. Besides directly regulating TG2 levels, CHIP can antagonize the activity of TG2 by conversely regulating several pathways activated by TG2. For instance, TG2 activates non-canonical NF-κB signaling in multiple cancers [108], whereas CHIP downregulates NF-κB-mediated signaling in colorectal cancer [101]. Moreover, CHIP can ubiquitylate Akt and inhibit the PI3K/Akt/mTOR signaling [109], while TG2-mediated degradation of PTEN activates the same pathway [95]. HIF-1α levels can also be downregulated by CHIP through direct ubiquitylation [96], when TG2 leads to upregulation [53] and accumulation [74] of HIF-1α. Thus, high TG2 and low CHIP levels in RCC may result in TG2 mediated activation of opposing signaling pathways by compromising the cellular degradation mechanisms.

## 5. Transglutaminase 2-Dependent Metabolic Switch in Renal Cell Carcinoma

Dysregulation of metabolic pathways is an important aspect of RCC [110]. HIF-1α plays a key role in the induction of glycolysis, as the oncogenic modulations leading to HIF-1α stabilization can promote alterations in cancer cell metabolism. In addition to the depletion of VHL, the accumulation of Krebs cycle substrates can lead to normoxic HIF-1α stabilization in RCC. Germline mutations in fumarate hydratase (FH), which is responsible for the conversion of fumarate to malate, results in the accumulation of fumarate in patients with hereditary leiomyomatosis RCC (HLRCC) [111,112,113]. Similarly, alterations in genes encoding succinate dehydrogenase (SDH), an enzyme that converts succinate to malate in Krebs cycle, are associated with the accumulation of succinate in familial kidney cancer patients. In renal tumors associated with the loss of FH or SDH activity, the accumulated fumarate and succinate molecules inhibit HIF prolyl hydroxylases (HPH), which is responsible from the hydroxylation of HIF-1α, and hence, its recognition by VHL. Inhibition of HPH enzymes thus leads to the accumulation of HIF-1α, which can upregulate several genes involved in the aerobic glycolysis, including glucose transporter 1, phosphofructokinase, pyruvate dehydrogenase, and lactate dehydrogenase [114]. Moreover, activation of the PI3K/Akt/mTOR pathway known to stimulate glycolysis directly by upregulating several glycolytic enzymes can also lead to normoxic HIF-1α accumulation in RCC [115]. A potential consequence of TG2 overexpression in RCC might therefore be of relevance to cell metabolism through the modulation of glycolytic metabolism at VHL/HIF-1α and PI3K/Akt/mTOR signaling axes. In fact, TG2 overexpression was found to deplete aconitase 2 enzyme in Krebs cycle, inducing a shift to the glycolytic state in RCC [116].

## 6. The Prognostic Value of Transglutaminase 2 in Renal Cell Carcinoma

The potential prognostic importance of TG2 in cancer was first recognized with sequential studies on tumor tissues and cell lines of breast cancer, showing elevated levels of TG2 in correlation with drug resistance [31,108]. Subsequently, association of TG2 overexpression with aggressive form of the disease was reported for pancreatic [30,117], glioblastoma [118], melanoma [32], lung [34], ovarian [33,119], colon [120] cancers and most recently, RCC [29,35,36]. Analysis of 95 primary RCC tumors collected by radical nephrectomy showed that the simultaneous upregulation of TG2 with its cell surface binding partners ITGB1 and SDC4 increased the risk of developing metastases by 3-fold. In agreement with this, both primary (Caki-2 and A-498), and metastatic (Caki-1 and ACHN) RCC cell lines exhibited higher transcriptional expression of TG2, while only the metastatic cells showed a concurrent upregulation of ITGB1 and SDC4. In association with ITGB1 and SDC4, TG2 was shown to act as an adhesion protein triggering integrin-mediated survival signaling; hence, elevated TG2 levels might lead to the metastatic potential through activation of mitogenic pathways [29]. Immunohistochemical analysis of tissue sections of both primary and metastatic sites showed that TG2 expression of primary site tumor samples from metastatic RCC patients was significantly higher compared to that of non-metastatic RCC patients. In contrast, the primary and metastatic site tumors from mRCC patients failed to display a difference in terms of TG2 levels. It was suggested that the elevated expression of TG2 in primary site tumors might occur during the initial tumor growth to enhance cell adhesion. Moreover, Kaplan-Meier analysis showed that elevated TG2 expression was correlated with a decrease in 5-year disease-free survival [36]. In a following study on radical and partial nephrectomy specimens from 638 ccRCC patients, a small number of samples exhibiting significantly higher TG2 expression was found to be correlated not only with a high metastatic potential but also with the high nuclear grade and worse prognosis [35]. Recently, a retrospective study on RCC tumor microarray using immunohistochemistry was performed to consider the prognostic value of TG2 along with proteins important in the regulation of transcription, cell cycle, growth signaling and apoptosis, as well as DNA damage and chromatin dynamics. Based on the immunohistochemical staining results, TG2 was only found to be related to disease progression in terms of invasion, metastasis, and therapeutic resistance [121].

## 7. Transglutaminase 2-Mediated Adhesion/Migration and Cancer Stemness in Renal Cell Carcinoma

Given that elevated TG2 expression has been associated with drug resistance and evasion of apoptosis in several cancers including RCC, the therapeutic potential of TG2 inhibition has been evaluated in various studies. TG2 knockdown, as well as inhibition of its enzyme activity, has been reported to increase the cancer cell sensitivity to drugs, potentiating apoptosis [117,122,123,124,125,126,127]. Despite being considered as a putative gene in the emergence of multiple drug resistance, the exact mechanism underlying TG2-mediated resistance in cancer has not been completely elucidated. It was observed that TG2-induced integrin-mediated survival signaling was responsible for the resistance of melanoma cells against cisplatin and dacarbazine [32], and lung cancer cells against doxorubicin [125]. It was also reported that high levels of TG2 in glioma cells conferred resistance preferentially against doxorubicin, while failing to respond to several other chemotherapeutics, indicating that TG2-mediated resistance can be drug-specific [128].

One potential mechanism of TG2-mediated drug resistance may rely on the c-Src-mediated activation of PI3K/mTOR survival signaling [129,130]. Transglutaminase 2 was shown to form a complex with c-Src and PI3K, facilitating the phosphorylation of the p85 regulatory subunit of PI3K by c-Src, which resulted in the activation of p110 catalytic subunit of PI3K and its downstream effectors in mTOR pathway [129]. This could represent an alternative TG2-dependent mechanism for the activation of mTOR pathway in RCC, in addition to the depletion of p53 and PTEN through degradation pathways.

On the other hand, TG2 overexpression is frequently associated with constitutive activation of NF-κB, which can regulate cell proliferation, survival, and metastasis. TG2 was suggested to potentiate cancer metastasis by inducing EMT through NF-κB activation, which results in the acquisition of a stem cell-like phenotype [53,108,131]. The ability of TG2 to foster stem cell phenotypes by facilitating anchorage-independent growth was shown in breast, ovarian, squamous carcinoma, and glioma cells [53,61,131,132]. The so-called cancer stem cells (CSCs) can be identified by the preferential expression of specific cell surface antigens, depending on the type of cancer. A tumor-initiating CSC population in RCC has been defined by the expression of CD105, CD44, and CD73 cell surface markers [133], and TG2 was found to be necessary for maintenance of the CD105+/CD44+/CD73+ phenotype in metastatic Caki-1 RCC cell population [134]. In support, TG2 expression was shown to potentiate the expression of EMT-inducing transcription factors Zeb1, Snail1, Snail2, Twist1 and Twist2, as well as EMT markers N-cadherin and vimentin in mouse RCC cell line RenCa [55].

Aside from modulating the pathways related to cancer cell growth, proliferation, and maintenance, TG2 can regulate the cytoskeletal organization, adhesion, migration, and invasion of cancer cells [135,136]. TG2 potentiates integrin clustering on the cell surface and activates ROCK kinase, the modulator of actin stress fibers, through inactivation of RhoA GTPase in the upstream [137]. Intracellularly, TG2 colocalizes with actin in the protruding ends of migrating cells, driving actin polymerization and regulating the cells’ ability to adhere and migrate [136]. It is now known that TG2 can also operate with ITGB1 and SDC4 in the organization of cell adhesion and migration [30,138,139]. The silencing of TG2 expression in primary site A-498 and Caki-2, and metastatic site Caki-1 and ACHN RCC cell lines was found to cause a disruption in the organization of actin cytoskeleton organization, as well as a reduction in the attachment and spreading of all cell lines on ITGB1 substrates [134]. The migration potential of Caki-1, Caki-2, and ACHN cells were hindered significantly upon TG2 downregulation. These results are in support of earlier observations implicating TG2 in RCC tumor aggressiveness and higher pathological grades associated with poor prognosis and disease-free survival [29,35,36].

## 8. Conclusions

Renal cell carcinoma (RCC) is a prevalent cancer disease that still requires the development of efficient therapeutic strategies to provide long-term disease-free survival of patients [41,42,43]. The identification of molecular targets that contribute to RCC oncogenesis has been an intensive area of research [48]. Accumulating evidence has suggested TG2 as an important target in RCC biology [29,34,36,49,55,85,93,107,116]. Transglutaminase 2 expression is generally low in primary tumors while its level is increased in metastatic and drug-resistant tumors. As a potential prognostic marker [29,35,36], elevated TG2 expression has been strongly associated with RCC progression in terms of cell survival, invasion and migration [55,134].

The existing controversy on the role of TG2 in cancer progression makes it difficult to develop efficient therapeutic strategies. Whether TG2 acts as a tumor suppressor or oncoprotein seems to depend on the cell type and context [140]. Inhibition of TG2 has been shown to increase drug-induced apoptosis in several cancer cell lines and evaluated as a sensitizer to chemotherapy in preclinical animal models of breast, ovarian, non-small cell lung, melanoma, colon cancers, glioblastoma, and meningioma [117,122,123,126,127,141]. Use of TG2 specific inhibitor GK921 and streptonigrin as monotherapeutic strategy in RCC mouse xenograft models showed a strong anti-tumor effect by stabilizing the cellular p53 levels and inducing apoptosis [93].

**Figure 1:**
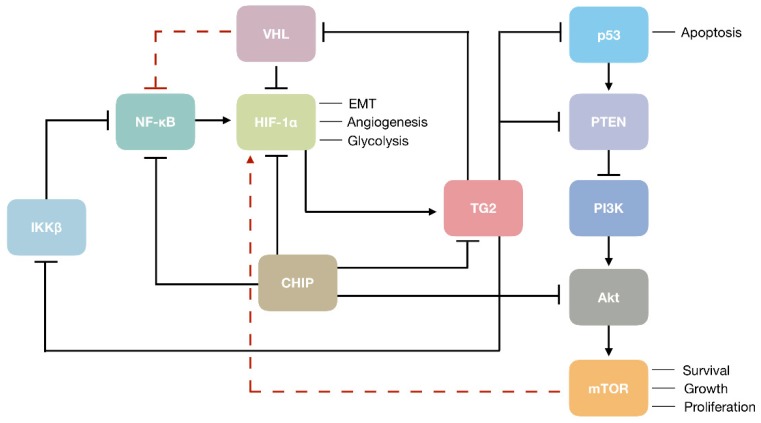
Transglutaminase 2 (TG2) in the modulation of renal cell carcinoma (RCC) oncogenesis. Black arrows and blunt ends show activation and inhibition of target molecules, respectively. Dashed red lines indicate an indirect mechanism of regulation.

Transglutaminase 2can contribute to the aggressive form of RCC through the modulation of several important mechanisms. For instance, TG2 can increase the intracellular level of HIF-1α in RCC cells by depleting its negative regulator VHL [74]. Transglutaminase 2 may also increase HIF-1α expression directly through the noncanonical activation of NF-κB, the upstream transcription inducer of HIF-1α [53]. Being one of the key oncogenic molecules in RCC, HIF-1α is responsible for regulating the expression of genes involved in EMT, angiogenesis, and glycolysis, thence TG2-mediated activation of HIF-1α may explain the promotion of EMT [55] and metastatic phenotype [134] by TG2 in RCC. (Figure 1). On the other hand, TG2 may act as a prometastatic protein in RCC by mediating cell adhesion and migration through its association with integrin and syndecan cell surface receptors, inducing the formation of focal adhesions and actin stress fibers [134]. Transglutaminase 2 may also contribute to the RCC progression by the downregulation of tumor suppressors associated with the RCC development, including p53 and PTEN [85,91,92,95]. In RCC cells, TG2-mediated depletion of p53 by autophagy was shown to render cells resistant to apoptotic stress [85]. As the transcription of PTEN can be activated by p53 [142], TG2-mediated depletion of p53 can result in the suppression of PI3K/Akt/mTOR signaling pathway by PTEN (Figure 1). In a more direct fashion, TG2 was shown to downregulate PTEN by protein-protein interaction, leading to its ubiquitin-dependent degradation [95,117]. Taken together, TG2 may act as a maestro in the orchestration of key oncogenic mediators in the complex setting of RCC, and may represent a promising target due to its contribution to disease progression, both as a main player and a pawn.
